# Targeted inhibition of tumor-derived exosomes as a novel therapeutic option for cancer

**DOI:** 10.1038/s12276-022-00856-3

**Published:** 2022-09-18

**Authors:** Ye Li, Zhuo-Kun Chen, Xu Duan, He-Jing Zhang, Bo-Lin Xiao, Kui-Ming Wang, Gang Chen

**Affiliations:** 1 The State Key Laboratory Breeding Base of Basic Science of Stomatology (Hubei-MOST) & Key Laboratory of Oral Biomedicine Ministry of Education, School and Hospital of Stomatology, Wuhan University, Wuhan, PR China; 2 Department of Oral and Maxillofacial Surgery, School and Hospital of Stomatology, Wuhan University, Wuhan, PR China; 3 TaiKang Center for Life and Medical Sciences, Wuhan University, Wuhan, PR China; 4 Frontier Science Center for Immunology and Metabolism, Wuhan University, Wuhan, PR China

**Keywords:** Targeted therapies, Cancer microenvironment

## Abstract

Mounting evidence indicates that tumor-derived exosomes (TDEs) play critical roles in tumor development and progression by regulating components in the tumor microenvironment (TME) in an autocrine or paracrine manner. Moreover, due to their delivery of critical molecules that react to chemotherapy and immunotherapy, TDEs also contribute to tumor drug resistance and impede the effective response of antitumor immunotherapy, thereby leading to poor clinical outcomes. There is a pressing need for the inhibition or removal of TDEs to facilitate the treatment and prognosis of cancer patients. Here, in the present review, we systematically overviewed the current strategies for TDE inhibition and clearance, providing novel insights for future tumor interventions in translational medicine. Moreover, existing challenges and potential prospects for TDE-targeted cancer therapy are also discussed to bridge the gaps between progress and promising applications.

## Cancer therapy: Interrupting messages sent by tumors

Inhibiting or removing tumor-derived exosomes (TDEs), tiny membrane-bound packets of DNA, RNA, and proteins secreted by tumors, may improve cancer therapies. TDEs can suppress the body’s immune response, promote tumor progression and spread, and reduce efficacy of cancer drugs and immunotherapy. Gang Chen at Wuhan University, China, and co-workers have reviewed ways to remove or inhibit production of TDEs. They report that disruption of the genes for production of TDEs, drugs that inhibit TDE secretion, and removal of TDEs via plasma exchange or dialysis are all being investigated and show promise for reducing patient TDE load, thereby increasing the efficacy of anti-cancer drugs and immunotherapy. Future challenges include reducing side effects and finding less invasive ways to filter out TDEs. Gaining a better understanding of TDEs may help to improve therapies for many types of cancer.

## Introduction

Since the discovery of exosomes in 1983, a new method of cell-to-cell communication was introduced to extend our perspectives of numerous physiological and pathological processes^1,2^. Exosomes are specific extracellular vesicles generated from the endosomal system instead of outward budding, typically ranging in size from 30 to 150 nm. Exosomes contain a large number of active constituents (e.g., proteins, lipids, and nuclear acids) and are considered key mediators of intercellular transportation^3,4^. Exosomes can be secreted by multiple kinds of donor cells, among which tumor cell-derived exosomes (TDEs) attract the most interest since they are involved in a series of critical functions, such as tumor growth and metastasis^5,6^.

**Fig. 1:**
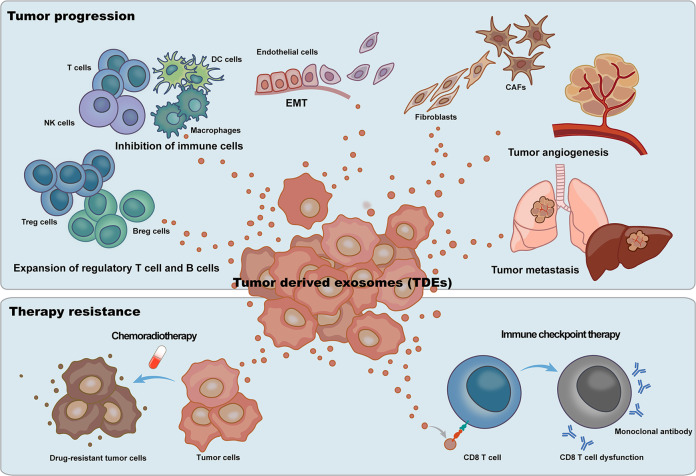
Tumor-derived exosomes (TDEs) play critical roles in tumor progression and impede tumor therapy. TDEs promote the expansion and activation of regulatory T cells and B cells and inhibit the function of effector T cells and NK cells to create immunosuppressive tumor microenvironments. By not only reprogramming normal fibroblasts into CAFs but also inducing CAFs to transform epithelial cells to a mesenchymal phenotype, TDEs also induce tumor progression and metastasis. TDEs mediate the activation of endothelial cells, leading to tumor angiogenesis. For tumor therapy, TDEs mediate chemotherapeutic drug resistance phenotypes in tumor cells by delivering multidrug transporters. In addition, TDE impedes the effect of tumor immunotherapy through the contained immune checkpoints, especially PD-L1.

Accelerating evidence suggests that ubiquitous TDEs in the tumor microenvironment (TME) play critical roles in tumor progression (Fig. 1). Transferring biological information locally and distantly, TDEs regulate the fate of their target cells through autocrine and paracrine pathways^7,8^. TDEs communicate with tumor cells, immune cells, cancer-associated fibroblasts (CAFs), and host vasculature in the TME and from a distance^9,10^. It has been indicated that TDEs can be taken up by tumor cells and inhibit the further release of TDEs, forming a negative feedback loop regulation. Moreover, TDEs also modulate the function of recipient tumor cells in proliferation and metastasis^11,12^. Immune cells are critical antitumor effectors in the TME^13,14^. Suppressing T-cell proliferation and inhibiting CD8 T-cell activation, TDEs contribute to immune escape^15,16^. In addition, TDEs may also induce T-cell differentiation into a suppressive regulatory T-cell (Treg) phenotype, favoring immunosuppression^17–19^. Producing immunoglobulins and presenting antigens, B cells play critical roles in cancer immunity. Regulatory B cells (Bregs) can be induced by TDEs, facilitating immune tolerance^20,21^. In addition, TDEs also present suppressive effects on natural killer cells^22,23^, dendritic cells^24–26^, and macrophages^27–29^ in the TME, promoting tumor progression and benefiting immune escape^30^.

CAFs constitute the major cellular component in solid tumors. Distinct from normal fibroblasts, CAFs are able to secrete multiple proinflammatory factors that contribute to tumor growth and metastasis^31,32^. Tumor cells communicate with CAFs through TDEs. TDEs may reprogram normal fibroblasts into CAFs, mainly through the delivery of critical proteins and microRNAs (miRNAs) to activate the transforming growth factor-β (TGF-β) signaling pathway^33–35^. In addition, TDEs could also induce CAFs to transform epithelial cells to a mesenchymal phenotype, promoting tumor metastasis^36^. TDEs accelerate angiogenesis in the TME to construct a new blood vessel network for tumor progression^37,38^. Containing vascular endothelial growth factor (VEGF) and other critical modulators in angiogenesis and targeting endothelial cells (ECs), TDEs reprogram ECs and activate angiogenic signaling pathways, inducing neovascularization in the TME^39–41^. In addition, ligand/receptor-mediated interactions are also involved in TDE-induced angiogenesis of ECs^42^.

In addition to the critical roles of TDEs in tumor progression, TDEs also contribute to drug resistance and impede the effective response to antitumor immunotherapy^43,44^. Accelerating evidence indicates that TDEs contain a large number of nucleic acids that may transition drug-sensitive cancer cells to a resistant phenotype^45,46^. In addition, TDEs deliver P-glycoprotein (P-gp), an ATP-dependent multidrug transporter, in an autocrine way to induce the extrusion of cytotoxic drugs^47^. Acquired drug resistance could also be achieved by the transmission of proto-oncogenes such as PTPRZ1-MET^48^. Checkpoint blockade immunotherapies targeting the programmed cell death-1 receptor (PD-1)/programmed cell death 1 ligand 1 (PD-L1) axis have emerged as promising treatments for cancer patients^49^. Nevertheless, the response rate is not satisfactory and partially due to the adaptive resistance mechanism mediated by TDEs^50,51^. Carrying PD-L1 inherited from their donor cells, TDEs bind to immune cells through the PD-1/PD-L1 axis, leading to the dysfunction of antitumor effectors^52–54^. Studies also suggested that PD-L1 on TDEs could directly bind to anti-PD-L1 antibody and may lead to immunotherapy resistance^55^.

Due to their involvement in tumor progression and therapy resistance, TDEs need to be inhibited to improve the prognosis of cancer patients. In the present review, we systematically overviewed the current strategies for TDE inhibition and clearance and suggested opportunities for tumor interventions in future translational medicine. Moreover, existing challenges, as well as potential prospects for TDE-targeting cancer therapy, are also discussed to bridge the gaps between TDE inhibition and the promising future of cancer therapy.

## TDE biogenesis and secretion

**Fig. 2:**
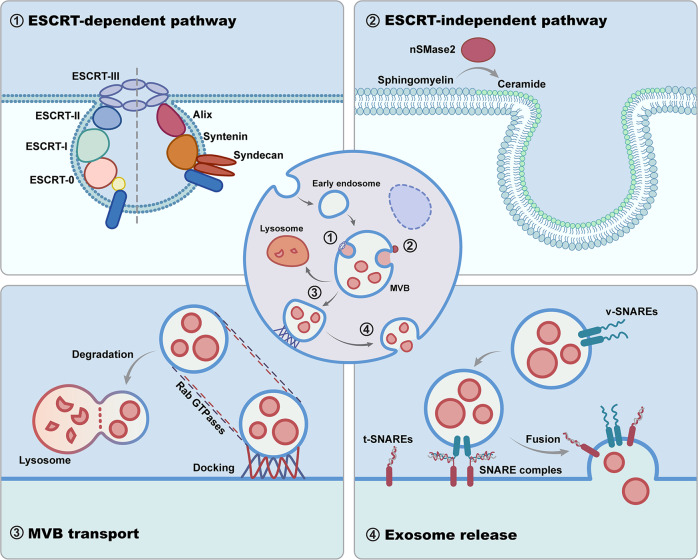
Critical modulators involved in TDE biogenesis and secretion. The biogenesis of TDEs begins with early endosomes budding inward to form the MVB, which depends on ESCRT complexes or through the ESCRT-independent pathway based on nSMase2. Subsequently, MVBs fuse with lysosomes for degradation or are docked to the cell periphery with the favor of Rab GTPases for secretion. Finally, SNARE complexes drive membrane fusion for TDE release.

**Fig. 3:**
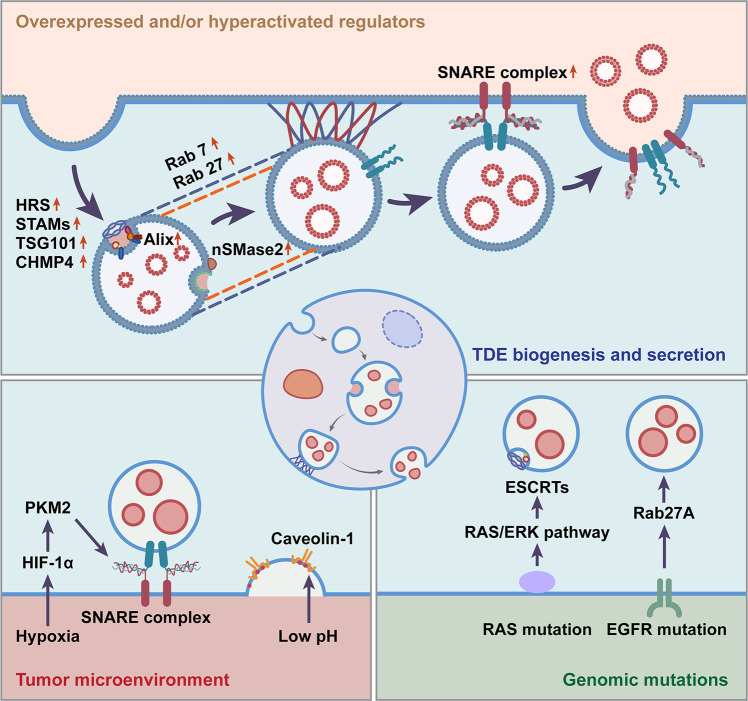
Exclusive machinery for TDE biogenesis and secretion. Key regulators of exosome biogenesis are overexpressed and/or hyperactivated in various tumor cells, contributing to the aberrant secretion of TDEs. The tumor microenvironment (TME) benefits the biogenesis and secretion of TDEs in multiple manners. Under hypoxic and acidic conditions, the biogenesis and secretion of TDEs are crucially regulated, making them different from non-TDEs. Due to specific genomic mutations (e.g., RAS and EGFR) of tumor cells, there are distinguishing mechanisms underlying the biogenesis and secretion of TDEs compared with non-TDEs.

As an important subset of exosomes, TDEs share common mechanisms of biogenesis with non-TDEs (Fig. 2) that begin with the inward membrane budding of early endosome (EE) to form multivesicular bodies (MVBs)^56^. It is well known that the endosomal sorting complex required for transport (ESCRT) machinery pathways are critically involved in the mechanism of TDEs. The ESCRT system consists of four complexes (named ESCRT-0, ESCRT-I, ESCRT-II, ESCRT-III) and accessory components^57,58^. The early ESCRTs (ESCRT-0 and ESCRT-I) are more responsible for cargo sorting. Initially, the ESCRT-0 complex binds phosphatidinositol-3-phosphate (PI3P) on the EE. Then, ESCRT-I and ESCRT-II are recruited and aggregated on the endosomal membrane. With high affinities, the rigid polyvalent membrane binding structures of ESCRT-I and ESCRT-II promote early endosomal membrane deformation and inward budding to form a narrow membrane neck. ESCRT-III is subsequently recruited to cleave the neck, forming the intraluminal vesicles (ILVs) of MVB^58^. TDEs are consequently released by the fusion of MVBs with the tumor cell plasma membrane^59^. Studies have reported that most of the key regulators, for example, ESCRT-0 proteins (HRS, STAM1, STAM2)^60–62^, ESCRT-I protein TSG101^63^, ESCRT-III proteins (CHMP4A, CHMP4B, CHMP4C)^64^, and accessory protein Alix^65^, are overexpressed and/or hyperactivated in various tumor cells, contributing to the aberrant secretion of TDEs (Fig. 3). In this context, although the mechanism is shared, the biogenesis and secretion activity of TDEs are different from those of non-TDEs, owing to aberrantly expressed regulators.

Colombo et al. revealed that ESCRT-0/I (HRS, STAM1, and TSG101) knockdown critically inhibited TDE secretion by HeLa-CIITA cells^66^. In addition to abundant numbers, the function of TDEs strongly depends on the encapsulated critical protein cargos, such as PD-L1^55^. In this context, fully revealing the mechanism of cargo sorting would provide new insights for TDE inhibition. ESCRT-0 is generally considered the driver responsible for sorting ubiquitinated protein cargo into ILVs. Bache et al. revealed that HRS, STAM proteins and EPS15 formed a multivalent complex, which had multiple ubiquitin-binding domains to cluster ubiquitin cargos on the endosomal membrane^67^. Deletion of certain ESCRT subunits leads to changes in the protein composition of TDEs. In our previous study, when HRS was downregulated in malignant melanoma cells, a decrease in the level of exosomal PD-L1, as well as an increase in cellular PD-L1, was achieved, suggesting HRS as a potential target for functional cargo sorting of TDEs^52^. Further elucidation of critical TDE cargo sorting regulated by ESCRT-0 would be beneficial for functional TDE inhibition.

Baietti et al. indicated that the Syndecan-Syntenin-Alix axis was crucial for TDE biogenesis and cargo sorting manipulation in breast cancer cells^68^. Syndecan is a ubiquitinated transmembrane protein that is supplied with heparan sulfate on the membrane. Alix binds to syntenin, the cytoplasmic adaptor of syndecan, regulating the biogenesis of ILVs. Recent evidence demonstrated that Alix also recruited ESCRT-III proteins to regulate TDE biogenesis and specific cargo sorting, such as tetraspanins, independent of other ESCRT subunits^69,70^. Being able to bind both ubiquitinated and nonubiquitinated proteins, Alix may be considered a potential target for TDE inhibition. However, Monypenny et al. indicated that Alix depletion resulted in defective PD-L1 trafficking through MVBs. Loss of Alix promoted PD-L1 redistribution to the cell surface and conferred an EGFR-dependent immunosuppressive phenotype^71^. Taken together, future studies should take combinations between TDE inhibition and the related effects into consideration to achieve effective TDE-targeted cancer therapy.

It has also been found that the lipid raft microdomains distinctly segregated protein cargos on the endosome membrane^72,73^. Neutral sphingomyelinase 2 (nSMase2) is able to hydrolyze the sphingomyelin of the endosome membrane into conical ceramide, resulting in negative membrane curvature by its cone-shaped structure. Subsequently, the endosome membrane bends toward the inner cavity and sorts the lipids and protein cargos into the ILVs^73^. When nSMase2 was knocked out in the PC3 prostate cancer cell line, the secretion of TDEs was critically inhibited^53^.

Before the release of TDEs, secretory MVB is transported to the cell periphery and docked to the plasma membrane. The Rab GTPase family contributes to the underlying mechanism of this trandport^74,75^. It has been noted that the 2 isoforms of Rab27, i.e., Rab27a and Rab27b, are involved in membrane transport with distinguished roles. Specifically, Rab27a regulates the docking and fusion of MVBs with the plasma membrane, while Rab27b participates in membrane transfer to MVEs from the Golgi network (TGN)^76^. Rab27a or Rab27b knockdown reduces TDE secretion of various types of cancer cells, such as HeLa cervical cancer cells^76^ and T24 bladder cancer cells^77^. Rab7 has also been found to play a critical role in TDE secretion by MCF-7 human breast cancer cells^68^. Other Rab GTPases, such as Rab11 and Rab35, participate in exosome formation of human retinal pigmented epithelial 1 (RPE1) cells^78^, also indicating a potential role in TDE biogenesis. After MVB docking, the soluble N-ethylmaleimide-sensitive component attachment protein receptor (SNARE) complexes start to drive membrane fusion and subsequent TDE secretion. The SNARE complexes are formed by v-SNAREs on the secretory MVB and t-SNAREs on the plasma membrane^79–81^. It has been indicated that syntaxin 6^81^, YKT6^82^, and VAMP7^83^, all SNARE proteins, regulate the TDE secretion of C4-2B and CWR-R1 prostate cancer cells, A549 lung cancer cells and K562 leukemic cells, respectively.

In addition to universal regulation, the exclusive TME may also benefit the biogenesis and secretion of TDEs in multiple manners (Fig. 3). Hypoxia and low pH are the key features of the TME^84^. Under hypoxic and acidic conditions, the biogenesis and secretion of TDEs are crucially regulated, making them different from non-TDEs^85,86^. Mechanistically, hypoxia-inducible factor-1alpha (HIF-1α) promoted the expression of pyruvate kinase 2 (PKM2)^87^. PKM2 was responsible for the phosphorylation of Ser95 of synaptosome-associated protein 23 (SNAP-23), a critical component of the SNARE complex, thereby promoting the secretion of TDEs^88^. Low pH is also considered a key microenvironmental factor that regulates the biogenesis and secretion of TDEs. It has been elucidated that acidic conditions could promote the secretion of TDEs by enhancing the function of caveolin-1^86^, which is an important initiator of exosome biogenesis through the regulation of cholesterol contents.

Due to the specific genomic mutations of tumor cells, there are distinguishing mechanisms underlying the biogenesis and secretion of TDEs compared with non-TDEs (Fig. 3). RAS (H-RAS, N-RAS, and K-RAS) is the most frequently mutated oncogene in cancers^89^. Several key differences have been revealed between mutant RAS and wild-type RAS cell line-derived exosomes. Increased secretion levels and more oncogenic proteins were found in mutant K-RAS cell-derived exosomes^90^. Mechanistically, activation of the RAS/ERK pathway, as a result of RAS mutation, was associated with the ESCRT-dependent biogenesis and secretion of exosomes. In addition, mutation of epidermal growth factor receptor (EGFR), which is considered one of the characteristics of lung cancer, also leads to the increased biogenesis and secretion of TDEs^91^. Collectively, the aberrant levels of shared regulators, the exclusive TME and the specific genomic mutations may combine to contribute to the promoted secretion and altered molecular contents of TDEs.

## Inhibition strategies for TDEs

**Fig. 4:**
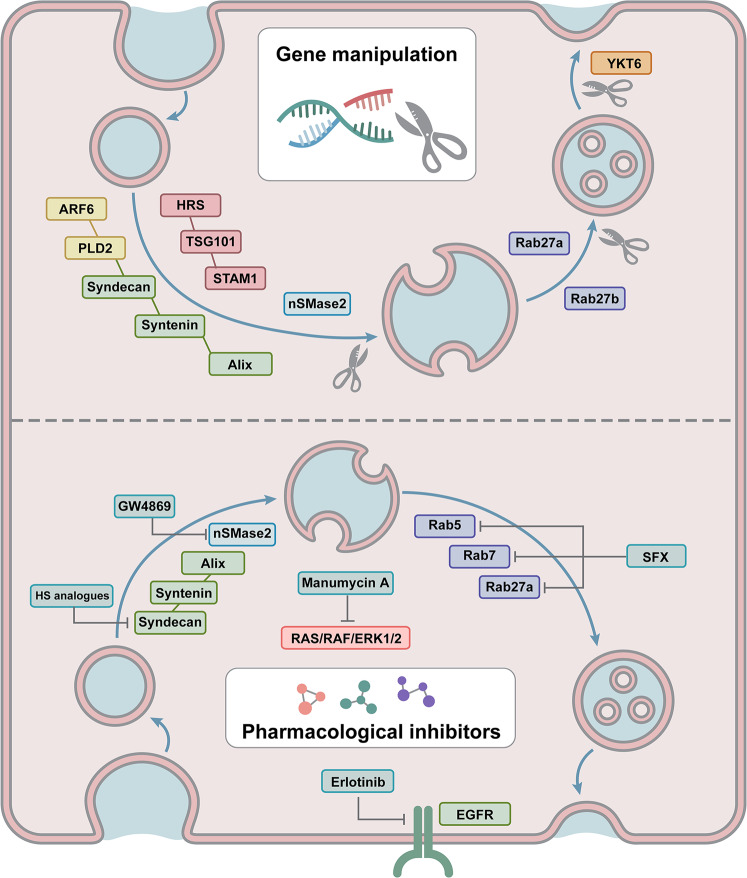
Emerging strategies employed for TDE targeted inhibition. Genetic manipulation has been proven to inhibit TDEs. With RNAi and the CRISPR‒Cas9 system to disrupt the genes that regulate TDE biogenesis and secretion, TDE inhibition was achieved. Pharmacological inhibitors have also been adopted to block TDEs by targeting critical regulators in TDE secretion.

Based on the mechanisms of TDE biogenesis and release, emerging strategies are employed for targeted inhibition of TDEs (Fig. 4 and Table 1). Genetic manipulation and pharmacological inhibitors are the most studied approaches.

**Table 1:** Inhibition strategies for TDEs.

Strategies	Targets	Cancer cell types/Recipients	References
Genetic manipulation			
RNAi	HRS	HeLa-CIITA cells, SCC61 cells, WM9 cells	^52,66,93^
	STAM1, TSG101	HeLa-CIITA cells	^66^
	Syndecan, Syntenin, Alix,	MCF-7 cells	^68^
	ARF6, PLD2	MCF-7 cells	^94^
	Rab27a/Rab27b	HeLa cells, T24 cells, SCC61 cells	^76,77,93,95^
	YKT6	A549 cells	^78^
Gene knockout	Rab27a and nSMase2	PC3 cells	^53^
Pharmacological inhibition			
GW4869	nSMase	MDA-MB-231 cells, T24 cells, A431 cells, SCC61	^77,93,98,99^
Manumycin-A Tipifarnib	FTases	C4-2B cells	^105^
			^106^
Ketoconazole	ERK signaling	RCC-24, 786-O, Caki-2	^107^
Erlotinib	EGFR	HSC‐4 cells	^108^
HS analogs	Syndecan-Syntenin-Alix	B16F10 cells	^109^
Sulfisoxazole	ETA	MDA-MB-231 and CT26 cells	^110,111^

### Genetic manipulation

Effective biotechnological tools, such as RNA interference (RNAi) and the CRISPR‒Cas9 system, have been widely used to reduce or completely disrupt the expression of critical genes involved in TDE biogenesis and secretion^92^. Colombo et al. took advantage of RNAi to silence 23 components of ESCRT machinery in HeLa-CIITA cells^66^. It was further indicated that the silencing of HRS, STAM1, or TSG101 led to a reduction in TDE secretion and exosomal MHC class II (MHC II). HRS has been shown to be involved in TDE biogenesis and cargo sorting in several studies. Hoshino et al. used RNAi to knock down HRS in SCC61 head and neck squamous cell carcinoma (HNSCC) cells and found that the secretion of exosomes was significantly reduced, as well as the protein contents, such as TSG101 and MT1-MMP, the critical matrix-degrading proteinase^93^. In our previous study, exosomal PD-L1 from malignant melanoma cells was found to contribute to immunosuppression. Encouragingly, when the expression of HRS was disrupted by RNAi, the level of exosomal PD-L1 decreased significantly^52^. Taken together, the above evidence suggests that HRS may be targeted for both TDE and effective cargo inhibition.

In MCF-7 human breast cancer cells, a portion of exosomes are formed through the Syndecan-Syntenin-Alix pathway. The disruption of Syndecan, Syntenin, or Alix by RNAi led to the reduced secretion of exosomes^68^. In another study, the biogenesis of syntenin-Alix exosomes was found to be regulated by the small GTPase ARF6 and its effector PLD2. Downregulation of ARF6 or PLD2 affected ILV formation and caused defects in MVB formation and subsequent exosome secretion^94^. In that case, ARF6 depletion did not implicate the specific cargo sorting of exosomes, which led to an aimless inhibition of both TDEs and non-TDEs.

Rab GTPases, which are responsible for intracellular vesicle transport, have also been used as targets for TDE inhibition. Evidence indicated that knockdown of Rab27a or Rab27b by RNAi inhibited the secretion of exosomes from a variety of tumor cells, such as cervical cancer cells^76^, bladder cancer cells^77^, HNSCCs^93,95^, etc. The downregulation of Rab27a or Rab27b blocked the transport of MVB to the cell periphery as well as the docking to the cell membrane, resulting in TDE inhibition^76^. Notably, Poggio et al. achieved the inhibition of exosomal secretion of PC3 prostate cancer cells by knockout of nSMase2 or Rab27a based on CRISPR‒Cas9, indicating a potential method of TDE inhibition^53^. However, since the deletion of nSMase2 resulted in only a partial loss of TDEs, more effective strategies or targets should be revealed in future studies. In addition, RNAi targeting Rab7 in MCF-7 breast cancer cells also inhibited the transport of MVB and led to reduced TDE secretion^68^. A recent study presented that Rab31 regulated exosome biogenesis in HeLa cells through an ESCRT-independent pathway and drove the sorting of protein cargos such as EGFR, providing a novel target for TDEs as well as cargo inhibition in future therapeutics^96^. For SNARE proteins that mediate the membrane fusion of MVB and cell membrane, Peak et al. found that downregulation of t-SNARE protein syntaxin 6 resulted in significantly reduced TDE secretion in C4-2B and CWR-R1 prostate cancer cells^81^. Ruiz-Martinez et al. also revealed inhibitory effects on TDE secretion in A549 cells by targeting YKT6^82^.

### Pharmacological inhibition

Over recent decades, pharmacological inhibitors have been extensively studied for suppressing TDEs, providing broad prospects in therapeutic applications. GW4869 is considered the most commonly used inhibitor of TDEs^97^. In both in vitro and in vivo cases, GW4869 inhibited the secretion of exosomes from a variety of tumor cells, including breast cancer cells^98^, bladder cancer cells^77^, epidermal cancer cells^99^, head and neck squamous cell carcinoma cells^93^, and malignant melanoma cells^100^, facilitating antitumor immunity. Yang et al. indicated that GW4869 inhibited TDEs from MDA-MB-231 breast cancer cells, resulting in a decrease in total protein content^98^. In a mouse model of breast cancer, GW4869 arrested tumor growth by inhibiting TDEs to promote antitumor effects, significantly enhancing the therapeutic effect of an anti-PD-L1 antibody. Given the hydrophobic nature of the GW4869 molecule, Wang et al. constructed a hyaluronic acid (HA)-based nanoplatform (HGF NPs) to deliver GW4869 in vivo^101^. The secretion of TDEs was dramatically decreased by HGFs assembled from GW4869 with a ferroptosis inducer (Fe^3+^), leading to the antitumor effect of cytotoxic T lymphocytes as well as long-lasting immunological memory. Although inhibiting roles on TDEs have been reported in numerous cases, the practical application of GW4869 is limited due to certain shortcomings. GW4869 is a blocker of nSMase2, which mediates the biogenesis and secretion of both TDEs and non-TDEs via an ESCRT-independent way^102^. Thus, direct application of GW4896 without tumor-targeted delivery may lead to nonspecific inhibition of both TDEs and non-TDEs. Additionally, considering that both ESCRT-dependent and ESCRT-independent machineries are involved in the regulation of TDE biogenesis and secretion^103^, application of GW4869 to inhibit the nSMase-mediated ESCRT-independent pathway may achieve only limited effects. In addition, GW4869 abrogates nSMase in a noncompetitive way^102^, which may further lead to limited efficiency of TDE inhibition. Finally, biosafety assessment of GW4869 is required before its clinical application because nSMase2 also contributes to multiple central biological processes^104^, thereby resulting in unpleasant side effects in vivo.

Based on the mechanisms that account for the biogenesis and secretion of TDEs, targeted inhibition strategies of TDEs were revealed. The genomic mutations that contribute to the aberrant biogenesis and secretion of TDEs could provide specific therapeutic targets. In this regard, Datta et al. conducted high-throughput drug screening and found a natural bacterial metabolite, manumycin-A (MA), which selectively affected RAS/RAF/ERK1/2 by targeting farnesyltransferases (FTases), resulting in the inhibited secretion of TDEs by castration-resistant prostate cancer (CRPC) cells^105^. In addition, tipifarnib, another FTase inhibitor, was also found to inhibit TDEs of prostate cancer cells, suggesting that FTase inhibitors can function as a class of potential effectors to block TDEs^106^. In a recent study, ketoconazole was adopted as an adjunctive therapy to enhance the efficacy of sunitinib in renal cell carcinoma treatment by inhibiting TDEs through downstream ERK signaling, providing updated evidence for the use TDE inhibitors as a novel option for tumor therapy^107^. Sasabe et al. employed erlotinib, an EGFR inhibitor, to suppress the negative effects of TDEs in oral squamous cell carcinoma (OSCC)^108^. This finding suggested that anti-EGFR agents may be effective for the treatment of cancer patients with EGFR mutations by not only blocking the EGFR signaling pathway but also attenuating the unpleasant roles of TDEs.

In addition to the specific inhibition of genomic mutations or exclusive TME, a shared regulatory mechanism could also be employed. Since most of the key regulators were found to be overexpressed and/or hyperactivated in tumor cells, they may serve as potential candidates for TDE inhibition. Wu et al. indicated that heparan sulfate (HS) analogs (heparin, low molecular weight heparin, and 6-O-desulfated heparin) specifically and efficiently inhibited TDE secretion by targeting Syndecan-Syntenin-Alix, leading to weakened tumor proliferation and invasion^109^. When B16F10 melanoma cells were treated with different HS analogs, both TDE secretion and protein cargo were inhibited. By screening 1163 drugs from FDA-approved libraries, Im et al. revealed that sulfisoxazole (SFX) selectively inhibited the secretion of TDEs from breast cancer cells^110^. SFX is generally employed as an oral antibiotic that is noncytotoxic at effective doses. By suppressing the transcription of Rab GTPases (Rab5, Rab7, and Rab27a) and ESCRT components (Alix, VPS4B), SFX inhibited the formation and secretion of MVB and induced their degradation within lysosomes, ultimately leading to TDE inhibition. In breast cancer xenograft mouse models, SFX presented significant antitumor and antimetastatic effects by inhibiting TDEs. Endothelin receptor A (ETA) was identified as the downstream effector of SFX, providing a potential target for TDE inhibition in breast cancer cells. In a recent study, SFX was found to be effective in reducing the level of circulating exosomes carrying PD-L1 in CT26 tumor-bearing mice, which reinvigorated the function of CD8 cytotoxic T cells and enhanced the efficacy of anti-PD-1 immunotherapy^111^. However, the specificity is limited since non-TDEs would also be inhibited, leading us to focus on tumor-targeted delivery of the drugs for improvement. Based on the key features of the TME (e.g., hypoxia and low pH), hypoxia- and/or pH-responsive drug delivery systems may be developed for targeted drug delivery. Taking lessons from well-designed TME-responsive systems, pharmacological inhibitors may be encapsulated and specifically delivered to tumor tissues, thereby achieving selective inhibition of TDEs.

## Clearance strategies for TDEs

In addition to the strategy based on genetic manipulation or pharmaceutical inhibition of TDEs in vitro and in vivo, Orme et al. pioneered the removal of TDEs from the circulation through therapeutic plasma exchange (TPE) in patients with malignant melanoma^112^. To discard the circulating exosomes, the plasma from patients was extracted by apheresis equipment and replaced by colloid solutions. With the diminished level of circulating TDEs, especially the critical cargos that modulate immunosuppression, the efficacy of immunotherapy may be improved.

Dialysis is also a commonly employed treatment to remove harmful substances from the circulatory system. As widely adopted therapies for kidney diseases, hemofiltration (HF) and hemoperfusion (HP) have also been developed to treat cancer. Taking advantage of a semipermeable membrane (diameter < 1 nm) to remove poison, an appropriately sized (diameter > 200 nm) microporous membrane would be promising for the clearance of TDEs. Although not yet applied in the clinic, affinity adsorbents have been adopted to selectively remove immunosuppressive cytokines, which may be a promising strategy for cancer treatment. It has been revealed that the cytokine network of the TME is involved in tumor progression and metastasis, leading Wang et al. further to utilize polyvinyl alcohol (PVA) microspheres coupled with heparin to remove tumor-induced cytokines^113^. The developed approach efficiently adsorbed immunosuppressive cytokines, such as VEGF and TGF-β, in the blood of tumor patients, facilitating cancer therapy. It is worth noting that PVA also presented promising biosafety, making it suitable for hemoperfusion in future translational medicine. Wu et al. also applied silica microspheres with a hemofiltration device to achieve the selective capture and removal of abundant circulating tumor cells as well as TDEs, providing potential choices for tumor therapy^114^. Recently, the rapid development of microfluid chips has paved the way for TDE filtration. Benefitting from the powerful compatibility and tiny size, microfluid chips assemble a large number of units with antibody coating for TDE elimination, which enables rapid and straightforward TDE clearance^115^.

## Current challenges and future perspectives

**Fig. 5:**
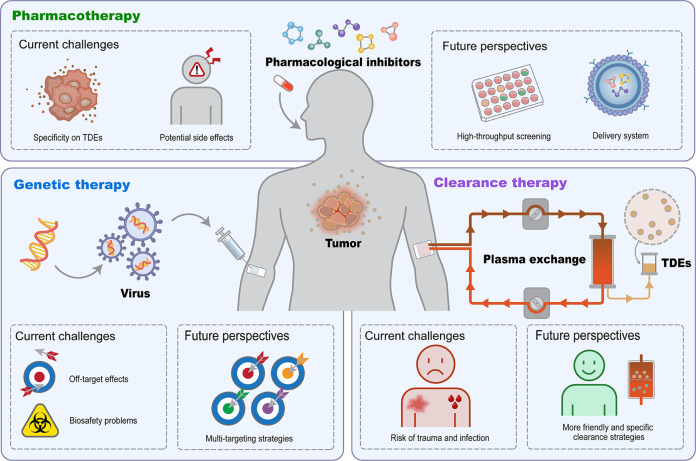
Current challenges and future perspectives for future eligible TDE inhibition and clearance to facilitate tumor therapeutics. Off-target effects and biosafety risks are the leading factors that limit the application of gene manipulation for TDE inhibition. Efforts should be devoted to developing an efficient and safe delivery system as well as targeting TDE-specific effectors. For promising pharmaceutical TDE inhibition, considerations on specific TDE blocking as well as controllable side effects should be taken ahead of translational medicine. The combination of high-throughput drug screening strategies and targeted drug delivery systems would promote the development of clinically available pharmacological inhibitor-based tumor therapies. For direct TDE clearance, the risk of trauma and infection as well as nonspecific elimination hamper the clinical translation to a large extent. In this regard, more friendly and specific strategies should be developed to pave the way for future clinics.

As outlined, approaches have been developed for the effective inhibition and clearance of TDEs. However, compared with the growing demand for scientific research and clinical application, more steps are needed (Fig. 5).

Despite its promising application in cancer research, TDE inhibition based on genetic manipulation still faces challenges in certain aspects^116,117^. First, off-target effects would result in unintended gene deletions, insertions, or mutations, leading to reduced block effect and inducing safety concerns^118^. Second, the specificity of the current inhibition strategy is limited. By blocking both TDEs and non-TDEs, potential adverse effects in tumor treatment were induced. Third, genetic modification may cause biosafety problems with virus-based systems, such as adeno-associated viral vectors or lentiviral vectors, resulting in uncertain virus‒host interactions such as severe immune reactions and cancer progression^119–121^. To obtain eligible TDE inhibition with genetic manipulation, efforts should be devoted to developing an efficient and safe delivery system as well as targeting TDE-specific effectors^122–124^. To this end, the mechanism of TDE biogenesis and secretion should be well recognized, providing more potential targets for future translation. With the understanding of the detailed mechanism of TDE biogenesis and secretion, multitargeting strategies may be developed to inhibit every critical step in TDE generation, leading to a whole pathway of TDE inhibition^53,125^. In addition, although the safety concerns of viral vectors have been dispelled in most in vitro and in vivo studies, considerations should be addressed on more alternatives in tumor therapy^126^.

For TDE inhibitors, serious considerations should also be taken ahead of translational medicine. First, it is difficult to target a single molecule with an inhibitor to effectively block TDEs since complicated pathways are involved in TDE biogenesis and secretion. On the other hand, with a heterogeneous population of exosomes in circulation, including but not limited to TDEs, obstacles also exist in specific inhibition of TDEs with the current strategies^2,127^. In these cases, multitarget pharmacological inhibitors should be developed to block as many pathways as possible that are critical for TDE biogenesis and secretion. Moreover, to avoid potential side effects to non-TDEs, precise release of TDE inhibitors should also be achieved. Considering that the exclusive TME (e.g., hypoxia and low pH) benefits TDE biogenesis and secretion, reshaping the TME or targeting the related downstream signaling pathways would provide new opportunities for precise TDE inhibition. It should also be noted that the development of clinically available pharmacological inhibitors is a time-consuming work with substantial costs^128,129^. In this regard, it would be more effective to screen TDE inhibitors with a high-throughput system. The quantitative analytical methodology should also be constructed for simultaneous screening with a wide range of candidate inhibitors^105,106,130^.

For direct TDE clearance, current strategies based on extracorporeal devices present limitations, such as invasion-induced trauma, bleeding risks and potential infections, hampering clinical translation. Moreover, the removal of total exosomes by blood purification would not only eliminate TDEs but also clear up the potential positive exosomes, leading to unknown effects that may further burden the tumor patients. In this case, more friendly and specific clearance strategies should be developed in future translation^112,131^. To this end, activating the in vivo phagocytosis system mediated by macrophages instead of developing a clearance system in vitro would be a beneficial approach^132^. Evidence has indicated that phagocytotic clearance by macrophages might be altered in the TME. The elevated PD-1 expression on macrophages in the TME was negatively correlated with phagocytic potency^133^. In this case, inhibiting or blocking PD-1 in macrophages in the TME might be an effective approach for activating phagocytosis-mediated clearance of TDEs. Furthermore, Lu et al. recently demonstrated that head and neck squamous cell carcinoma (HNSCC)-derived TDEs inhibited phagocytosis of macrophages through CD73, thereby triggering immune suppression and aggressive tumor growth^134^. Therefore, future studies may also attempt to block CD73 on TDEs to enhance the clearance of TDEs by phagocytes, thus facilitating tumor therapy.

## Conclusions

To conclude, TDEs play critical roles in tumor progression and mediate therapy resistance, leading to poor clinical outcomes. Taking lessons from the mechanism of TDE biogenesis and secretion, we discussed the emerging strategies for TDE inhibition and clearance, providing opportunities for future cancer therapy. To address the issues occurring in current inhibition approaches, considerations should be taken into account to achieve more specific and effective methods for genetic manipulation as well as pharmacological inhibition. Alternatively, friendly TDE clearance strategies should also be introduced. Taken together, targeted inhibition or clearance of TDEs may provide novel therapeutic options for future cancer treatment.
